# Medical Society of London

**Published:** 1896-11-14

**Authors:** 


### Medical Progress and Hospital Clinics.

[*The Editor will be glad to receive offers of co-operation and contributions from members of the profession. All letters should be addressed to* The Editor, at the Office, 28 & 29, Southampton Street, Strand, London, W.C.]

### MEDICAL SOCIETY OF LONDON.

At the meeting held on November 9th Mr. Lockwood showed a case of gastro-enterostomy for pyloric obstruction, due to a carcinoma of the pylorus with enlarged glands in the lesser omentum.

Three rows of sutures were inserted. The recovery was very complete, the patient being able to eat pretty much what he liked, taking beer and fish and cheese.

About the tenth day after the operation the sutures in the abdominal wound were removed, and shortly afterwards the patient, in coughing, forced out a loop of intestine. This, however, was returned, and kept in place by the application of strapping. This accident, however, was a lesson to him not to be in a hurry to take out the sutures in such a case. Although the operation lasted fifty minutes the patient did not suffer from shock, which Mr. Lockwood attributed to the fact that all the operation was done outside the abdomen, and that the patient was kept thoroughly warm all the time. The operation was done on January 13th, and the patient had remained very well since.

Mr. G. R. Turner showed a case of deformity of the lower jaw, said by the mother to be congenital. According to the father, however, it was not congenital, and did not commence till the patient was two years old, when he suffered from an attack of measles. The inferior maxilla was extremely ill-developed, being in all dimensions far too small to correspond with the upper jaw. The roof of the mouth was greatly arched. Mr. Bruce Clarke said he had never seen a case in which this deformity was bilateral as in this case, but he had seen cases in which the same sort of deformity had occurred on one side. In one case the tongue fell back and caused such serious symptoms that he had to perform tracheotomy to prevent suffocation. These cases seemed, as in this instance, to come on at from two to three years of age, and not to be congenital.

Mr. Clinton Dent showed a very extraordinary case of a man, twenty-nine years of age, who had suffered from *fragilitas ossium* from the earliest infancy. At four months old he had a fracture of the right femur, and altogether had had twenty-seven fractures of long bones. Of these eighteen had occurred in the lower extremities, ten on one side, eight on the other. All these fractures had united fairly well, but they had resulted in the most prodigious deformities. There was curvature of the spine and distortion of the ribs, both of which were increasing. His intelligence was good, and he occupied himself with watch making or watch cleaning. Several of his relations were affected with deformities. It might be of some interest to note that although he was breast-fed for four years the first fracture occurred when he was only four months old, long before the prolonged use of his mother's milk came into play as an etiological factor. Some very interesting X ray photographs of the affected parts were shown.

Dr. Walter Carr showed two cases of pseudo-hypertrophic paralysis, exhibiting the conditions met with at the two extremes of such cases. The first case was a girl, which, of course, is a very uncommon occurrence, most of these cases occurring in boys. She was five years old, and had shown symptoms for a year. The lordosis, the waddling gait, and the characteristic method of rising from the floor, were well shown. The calf muscles showed marked enlargement and hardening, as in a less degree did the infra-spinati. No muscles showed any wasting so far. The second case showed the other end—the last stage of the disease. Symptoms had existed for five years. There was extreme muscular atrophy, with contractures resulting in talipes equinus, and fixed flexion of the knees. Nevertheless, the calf muscles, the deltoids, and perhaps the infra-spinati, or such as was left of them, were hard. The wasting of the axillary folds was very marked. His younger brother was also affected with the same disease, for which he had been treated in the Homœopathic Hospital, the report of which hospital, containing illustrations of this case, was passed round.

Mr. D. H. Goodsall then showed a case of removal of pelvic tumour.

